# Ferroptosis in glioma treatment: Current situation, prospects and drug applications

**DOI:** 10.3389/fonc.2022.989896

**Published:** 2022-09-29

**Authors:** Yuhang Zhou, Chaoyou Fang, Houshi Xu, Ling Yuan, Yibo Liu, Xiaoyu Wang, Anke Zhang, Anwen Shao, Danyang Zhou

**Affiliations:** ^1^ Health Management Center, Tongde Hospital of Zhejiang Province, Hangzhou, China; ^2^ The First Clinical Medical College, Heilongjiang University of Chinese Medicine, Harbin, China; ^3^ Department of Neurosurgery, Shanghai General Hospital, School of Medicine, Shanghai Jiao Tong University, Shanghai, China; ^4^ Department of Neurosurgery, The Second Affiliated Hospital, School of Medicine, Zhejiang University, Hangzhou, China

**Keywords:** glioma, ferroptosis, targeting treatment, reactive oxygen species, iron metabolism

## Abstract

Ferroptosis is a regulatory form of iron-dependent cell death caused by the accumulation of lipid-based reactive oxygen species (ROS) and differs from apoptosis, pyroptosis, and necrosis. Especially in neoplastic diseases, the susceptibility of tumor cells to ferroptosis affects prognosis and is associated with complex effects. Gliomas are the most common primary intracranial tumors, accounting for disease in 81% of patients with malignant brain tumors. An increasing number of studies have revealed the particular characteristics of iron metabolism in glioma cells. Therefore, agents that target a wide range of molecules involved in ferroptosis may regulate this process and enhance glioma treatment. Here, we review the underlying mechanisms of ferroptosis and summarize the potential therapeutic options for targeting ferroptosis in glioma.

## Introduction

Glioma is the most common malignancy of the central nervous system (CNS) and manifests with highly invasive growth, neovascularization, and resistance to various combination therapies (1). Despite advanced therapeutic strategies, including aggressive surgery, radiotherapy, and chemotherapy, glioblastoma (GBM) patients still show poor prognosis and a median overall survival of less than 16 months (2). Despite aggressive treatment measures, including maximal safe surgical resection followed by external irradiation therapy accompanied with adjuvant temozolomide (TMZ) treatment, approximately 90% of grade WHO IV gliomas recur locally within 2 years (3). Gross total resection (GTR), defined as complete radiectomy of the contrast-enhanced region of high-grade glioma (HGG) and T2-weighted/fluid attenuated inversion recovery (T2/FLAIR) MRI-indicated hyperintensive nonenhancing lesions, almost always fails to completely remove all microscopic residual tumor cells (4). Similar to other malignancies, GBM exhibits a distinct anti-DNA-damage phenotype, which leads to chemoresistance (5).

Hence, therapies targeted to gliomas have not been considered sufficiently effective (6). However, ferroptosis has recently attracted considerable interest, especially because the mechanism involves downregulation and silencing of genes involved in the initiation and execution of cancer necroptosis (7). Ferroptosis is a unique iron-dependent form of nonapoptotic cell death in which the affected cells are morphologically, biochemically, and genetically distinct from apoptotic, necrotic, and autophagic cells (8). Ferroptosis is driven by the lost lipid repair enzymatic activity of glutathione peroxidase 4 (GPX4) and subsequent accumulation of lipid-based reactive oxygen species (ROS), particularly lipid hydroperoxides (9). As a common recognition feature, ferroptotic cells appear as clear and transparent round cells under the microscope, mainly composed of empty cytosol, which is called the “ballooning phenotype”. In addition, ferroptotic cells also have ultrastructural changes in mitochondria such as volume decreased, bilayer membrane density increased, outer mitochondrial membrane (OMM) destroyed, and mitochondrial cristae disappeared.

To promote tumor growth, cancer cells exhibit a higher iron demand than normal cells. This iron dependence makes cancer cells more susceptible to ferroptosis (10). Therefore, induced ferroptosis induction may offer the unique possibility of effectively eradicating certain tumor cells, especially those in a highly mesenchymal state (11) and those that evade drug treatment (12). Furthermore, ferroptosis plays a pivotal role in suppressing tumorigenesis by eliminating cells in environments that lack key nutrients or produce cellular stress or that are infected with pathogens (13). The ferroptotic sensitivity of cancer cells may be related to the activation of Ras-mitogen-activated protein kinase (MEK) (14), which contributes to the upregulation of transferrin receptor 1 and increased intracellular iron levels, as well as to the additional formation of ROS *via* inhibited cystine-based reactions (15). Many other molecules in different pathways have been found to be involved in ferroptosis in glioma (16), and the related content is summarized in this review.

## Focused overview of ferroptosis pathways

**Figure 1:**
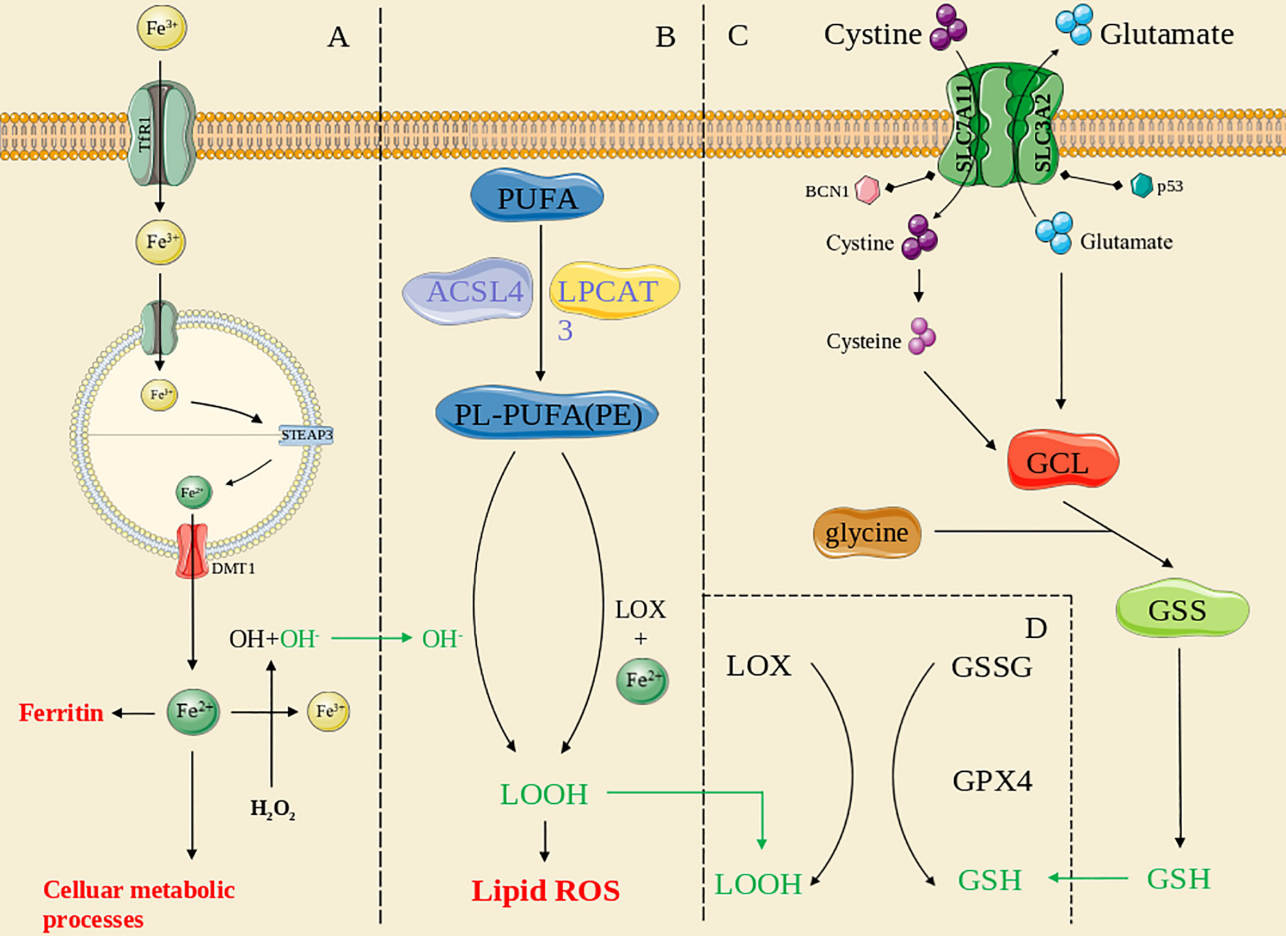
The overview of ferroptosis pathways. **(A)**: the iron metabolism pathway; **(B)**: the lipid peroxidation pathway; **(C)**: the antioxidant systems pathway; **(D)**: the GPX4-mediated pathway. The green line means the substance acts across pathways.

An overview of ferroptosis pathways is shown in **Figure 1**.

### Iron metabolism in ferroptosis

The regulatory mechanism that coordinates intracellular iron homeostasis is centered on iron regulatory proteins (IRPs), which exerts effects by binding to iron-responsive elements (IREs) (17, 18). Under physiological conditions, cellular iron absorption is controlled mainly by the plasma membrane protein transferrin receptor 1 (TFR1), and therefore, knocking down TFR1 expression can block transferrin-bound iron entry into a cell (19, 20), preventing ferroptosis caused by erastin or cystine deprivation (21). Diminishing ferritin expression (22) or FPN1 or ceruloplasmin depletion increases the cell sensitivity to ferroptosis (23, 24). In addition, reduced IRP2 activity, increased expression of transferrin (Tf) and the transferrin receptor (TFR) (19), and recognition of FTH1 by a specific cargo receptor (nuclear coactivator 4, NCOA4), which leads to formation of a complex that fuses with lysosomes (25), cause an abnormal increase in unstable intracellular iron stores, a critical factor in ferroptosis. Other iron metabolism-related proteins also affect cell sensitivity to ferroptosis (26), and certain genes exert the same effects. Recently, the critical role played by STEAP3 in cancer has been extensively investigated, and STEAP3 has thus been found to be a key regulator of ferroptosis by mediating iron metabolism (27, 28). Overexpression of STEAP3 contributes to iron uptake and maintains iron stores (29), supporting the proliferation of multiple types of cancer cells (30–32). Hence, dysregulation of iron metabolism is an important contributing factor to ferroptosis.

### Lipid peroxidation in ferroptosis

Lipids are critical for maintaining the membrane integrity of a cell, and extensive peroxidation of lipids changes the assembly, composition, structure, and dynamics of lipid membranes (33). Polyunsaturated fatty acids (PUFAs) containing phospholipids (PLs; PUFA PLs) are substrates for lipid peroxidation (34). ROS are free radicals and/or oxygen derivatives, including superoxide anions, hydrogen peroxide, hydroxyl radicals, lipid hydroperoxides, peroxy radicals, and peroxynitrite (35). Membranes containing high levels of PUFAs are extremely sensitive to ROS effects and highly vulnerable to lipid peroxidation (36, 37). Lipid undergo peroxidation through two routes: nonenzymatic autoxidation and enzymatic PL peroxidation; the former pathway is known as “nonenzymatic PL autoxidation”.

Nonenzymatic peroxidation of lipids is mediated by carbon- and oxygen-based radicals and can be divided into three discrete stages: initiation, proliferation, and termination (33). The initial phase involves a series of reactions collectively known as “Fenton chemistry” in which labile iron reacts with endogenous hydrogen peroxide or superoxide to form oxygen-based radicals (38). Radical compounds produce new radicals, which are markers of the proliferative phase. The hydroxyl and peroxide radicals produced through a Fenton reaction can form a resonant stable carbon-based radical by extracting hydrogen from the bis allylic methylene of a membrane PUFA, which can react with molecular oxygen in solution to form the lipid peroxide radical ROO^−^, which can remove a hydrogen from a different bis allylic methylene to generate peroxidized lipid (ROOH) and another carbon-based radical that can react with oxygen (33, 39, 40). Finally, antioxidants terminate radical propagation (41).

Enzymatic PL peroxidation is mainly mediated by cyclooxygenases (COXs), cytochrome p450 species (CYPs), NADPH oxidase (NOX), and, especially, lipoxygenases (LOXs) (42). Arachidonic acid (C20:4) and linoleic acid (LA; C18:2) are substrates for LOX (43), and ferric iron is a cofactor of LOX (44, 45). In contrast to 5-lipoxygenases, 12- and 15-lipoxygenases exhibit incomplete regional selectivity in producing lipid peroxides (46) and are thought to respond to intact phospholipids and do not promote hydrolysis for peroxidation (47, 48). Lipid hydroperoxides (LOOHs) and the autoxidation products of PUFAs are currently markers of ferroptosis (49, 50).

### Antioxidant systems in ferroptosis

In addition to lipid peroxidation, the cellular antioxidant system contributes to ferroptosis by decomposing ROS. GPX4 is a central factor in anti-ferroptosis reactions (51). This protein is expressed as several isoenzymes with different subcellular locations and distinct tissue-specific expression patterns (52, 53). GSH is a cofactor of GPX4, and GSH synthesis is maintained by the amino acid antiporter SLC7A11/xCT/system (54). Some small-molecule compounds can regulate the activity of glutamate-cysteine ligase (GCL) and xCT (8) and thus affect GSH synthesis, eventually leading to ferroptosis. Several other small-molecule compounds can directly inhibit GPX4 activity or cause GPX4 protein degradation (55, 56). Nonoxidized dopamine and activated heat shock protein family A member 5 (HSPA5) prevent GPX4 degradation (57, 58), whereas heat shock protein 90 (HSP90)-dependent chaperone-mediated autophagy promotes erastin-induced GPX4 degradation (59). Furthermore, GPX4-independent ferroptosis pathways have been identified. Ferroptosis inhibitory protein (FSP1) and CoQ10 facilitate a shuttle of reducing equivalents derived from NAD(P)H to the lipid bilayer (60). In addition, POR is involved in ML210-induced ferroptosis (61), and P53 can affect ferroptosis without GPX4 inhibition (62). The main regulatory factors are described in detail in the next section.

## Critical factors of ferroptosis in glioma

Ferroptosis follows multiple pathways and involves pivotal factors that are regulated by many different regulators. Certain regulators exert valuable regulatory effects and metabolic changes in glioma cells. In this section, the regulators best characterized to date are described, and additional regulators are presented in **Table 1**.

**Table 1:** Critical factors of ferroptosis in glioma.

Factors	Targets	Mechanism	Reference
GPX4	peroxide↓	affect LOX activity, reducing to peroxidation of PUFAs, inhibit ferroptosis	Seibt et al. (63)
	GSH	reduce LOOH, inhibit LPO, inhibit ferroptosis	Ursini et al. (64)
Nrf2	Keap1	dissociates from Keap1, interacts with ARE, maintain intracellular redox homeostasis	Zhang et al. (65)
	MRP1↑	prevents GSH efflux from the cells, strongly restrains ferroptosis	Cao et al. (66)
	xCT↑	reduced ROS formation, prevents ferroptosis	Fan et al. (67)
P53	xCT↓	combination with response elements in the xCT promoter region, inhibit its expression	Jiang et al. (68)
	USP7	promotes nuclear translocation of USP7, removes H2Bub1, reduces the expression of xCT	Wang et al. (69)
	SAT1	induces elevated ALOX15 levels, causes ferroptosis *via* oxidation of PUFA	Ou et al. (70)
BAP1	xCT↓	decrease H2Aub occupancy on the promoter and gene body of xCT	Zhang et al. (71)
OTUB1	p53	regulate the p53 pathway by regulating the activities of Mdm2 and Mdmx	Sun et al. (72)
			Chen et al. (73)
	xCT	Inactivation of OTUB1 lead to a substantial reduction in xCT levels	Liu et al. (74)
ATF4	xCT	ATF4 knockout will reduced xCT transporter activity	Dixon et al. (75)
			Chen et al. (76)
	ROS	ATF4 deficiency increases ROS levels	Angeli et al. (77)
NCOA4	iron homeostasis	iron-bound NCOA4 interacts with the ubiquitin E3 ligase HERC2, reduce the ferritinophagy	Mancias et al. (78)
	FTH1↓	decreased FTH1 levels would cause cells to respond to several ferroptosis-inducing agents	Hayashima et al. (79)
YAP/TAZ	Nuclear translocation	YAP/TAZ be phosphorylated by MOB1	Masliantsev et al. (80)
	autophagy↑	activated YAP/TAZ promotes autophagy, affects ferroptosis	Sun et al. (81)

The symbol ↓ means target factor level reduced, the symbol ↑ means target factor level rises.

### GPX4

GPX4, a core factor in the antioxidant system, regulates certain LOX activities by controlling cellular peroxide formation (82). LOX binds to molecular oxygen when iron is oxidized into trivalent iron and adds this molecular oxygen to a PUFA after proton extraction from the bis-allylic positions of the PUFA, leading to the enzymatic peroxidation of the PUFA (43). The GPX4-mediated antioxidant system can reduce the peroxide concentration, which may affect LOX activity, reducing the peroxidation rate of PUFAs and ultimately inhibiting ferroptosis (63). Studies have pointed out that 15-LOX and its linoleic acid (LA)-derived metabolites exerted protumorigenic effects on GBM cells *in vitro (* 83). This report may imply that GPX4 affects ferroptosis by regulating LOX activity and can be exploited for glioma treatment.

GSH is a reducing substrate for GPX4, and its interaction with SCL7A11 plays a crucial regulatory role in ferroptosis. However, both GSH and SCLA11 activities are intricately regulated by p53 and NRF2, among other proteins., as described in detail in a subsequent section (64).

Western blot and immunohistochemistry (IHC) analyses showed relatively high expression levels of Gpx4 in glioma tissues and cell lines, and its expression was found to be augmented as the glioma grade increased. In addition, experiments showed that knocking down GPX4 expression inhibited the proliferation and migration of glioma cells (84). Previously, inhibition of GPX4 activity was thought to induce apoptosis (85), and combined with the aforementioned findings, it can be concluded that GPX4 inhibition can also induce ferroptosis, which may become a new research target.

### Nrf2

Under normoxic conditions, Nrf2, a transcription factor, binds to Kelch-like ECH-associated protein 1 (Keap1) and is inactivated by proteasome degradation after ubiquitination (86). After cells contact a large number of electrophiles or cytotoxic agents or enter into an oxidative stress state, Nrf2 dissociates from Keap1 and rapidly transfers to the nucleus where interacts with antioxidant response elements (AREs) to ultimately maintain intracellular redox homeostasis (65). Nrf2 regulates ferroptosis by regulating the expression of genes related to GSH regulation (genes that encode proteins involved in GSH synthesis and, supply cysteine mediated by xCT, GSH reductase, GPX4), iron regulation (including export and storage of iron, heme synthesis, and catabolism), and NADPH regeneration (87–89). Considering recent research, we speculated that Nrf2 partially targets xCT to regulate GPX4 synthesis and function, thus regulating ferroptosis. When Keap1 activity is inhibited, Nrf2 activity increases, leading to the upregulated expression of the ATP-binding cassette (ABC)-family transporter multidrug resistance protein 1 (MRP1), which prevents GSH efflux from the cells and profoundly inhibits ferroptosis (66). The expression of Nrf2 was increased 3-fold in human GBM compared to that in normal brain tissue (67). Both the low expression of Keap1 and the overexpression of Nrf2 led to a significant increase in xCT mRNA levels (up to a 5-fold increase), which subsequently reduced ROS formation. In contrast, both the overexpression of Keap1 and the low expression of Nrf2 eventually led to a substantial increase in ROS levels (67). Thus, the levels of NRF2 are directly related to ferroptosis sensitivity, as increased NRF2 expression prevents ferroptosis, and decreased NRF2 expression enhances the sensitivity of cancer cells to ferroptosis (67, 90).

### P53

The tumor suppressor p53 is a transcription factor that regulates various cellular responses through selective transcriptional regulation of various target genes or interaction with other proteins. Studies have shown that xCT is a target of p53 and that p53 sensitizes cells to ferroptosis through transcriptional inhibition of xCT expression (68). The combination of p53 with response elements in the xCT promoter region inhibited xCT expression and increased the sensitivity of cancer cells to ferroptosis inducers such as erastin; however, p53^3RK^ failed to induce cell cycle arrest, senescence, or modulation and inhibited xCT expression, ultimately promoting the response to stress induced by ROS (68). However, another acetylation-defective mutant of p53, p534KR98 (with a lysine K98 substitution), showed no ability to reduce xCT expression (91). As recently reported, p53 sensitized cells to erastin-induced ferroptosis through a comprehensive pathway. P53 promotes nuclear translocation of USP7 (a deubiquitinase) that removes the H2Bub1 mark (monoubiquitinated histone H2B on lysine 120) from the regulatory region of the xCT gene. Loss of the H2Bub1 mark inhibited the expression of xCT, leading to ferroptosis (69).

Low-molecular-weight polyamines such as putrescine, spermidine, and spermine are amino acid-derived polycationic alkylamines involved in the regulation of cell growth, proliferation, and differentiation (92). Spermidine/spermine N1-acetyltransferase 1 (SAT1) is a rate-limiting enzyme that controls polyamine catabolism in cells by acetylating spermidine and spermine mediated through acetyl-coenzyme A (93). Overexpression of SAT1 causes a rapid depletion of spermidine and spermidine levels and an increase in putrescine abundance, which causes significant cellular growth inhibition and mitochondrial pathway apoptosis (94). SAT1 has been confirmed to be a transcriptional target of p53, and only the ferroptosis inhibitor ferrostatin-1 was able to inhibit ROS-induced cell death in SAT1-overexpressing cells. In contrast to its effect on conventional pathways, SAT1 exerted no effect on xCT or GPX4 expression or activity but induced an increase in ALOX15 level, which in turn led to ferroptosis mediated *via* the oxidation of PUFAs (70).

Glutamine metabolism affects ferroptosis and exerts a particularly high effect on serum-dependent pathways after amino acid deficiency (19). GSL2 (glutaminase 2) in mitochondria is a transcriptional target of p53 and is the core glutaminase in the glutamine-to-glutamate metabolic pathway (95). the GSL2 is transcribed by p53 and mediates the generation of GSH in LN-2024 cells (a human glioblastoma cell line) to enhance their antioxidant capacity (96).

In addition to the aforementioned effects, p53 inhibited ferroptosis in some tumor cells. For example, studies showed that binding of p53 to dipeptidyl peptidase-4 (DPP4) inhibited ferroptosis in colorectal cancer cells, and certain DPP4 inhibitors completely blocked erastin-induced cell death in p53-deficient colorectal cancer cells (97). These studies suggest that the inhibition of p53 activity is specific to ferroptosis inducers (98). The tumor suppressor CDKN1A/p21 induces cell cycle arrest and senescence (99, 100). Although the cell cycle arrest mediated by CDKN1A is insufficient to inhibit ferroptosis (101), the induction of p53 increases GSH synthesis and thus inhibits ferroptosis (102).

According to The Cancer Genome Atlas (TCGA) data, 78% of GBM cases present with mutations in the p53 pathway (103), including direct mutations in the p53 gene (in secondary GBM) and a loss of the INK4A/ARF (CDKN2A) gene locus, PTEN mutations and EGFR amplification/loss (in primary GBM) (104). Since p53 is involved in various cellular responses involving the cell cycle or leading to apoptosis, differentiation and DNA damage, the regulatory effect of p53 on ferroptosis needs to be assessed on the basis of the situation, and further research is required (105).

### BAP1

BRCA1-associated protein 1 (BAP1) is a tumor suppressor with functions such as tumor suppression, cell cycle control, DNA damage repair, and differentiation (106–109) that is widely recognized as a deubiquitinating enzyme (DUB) (110). Study results have suggested that wild-type (WT) BAP1 significantly decreased H2Aub occupancy on the promoter and gene body of xCT, but the C91A mutant did not exert this effect (71). Because WT BAP1 exhibited DUB activity and BAP1 C91A did not in this experiment, WT BAP1 was the clear cause of inhibited xCT expression (71). Therefore, BAP1 may be recruited by other proteins in the PR-DUB complex, such as ASXL1, which also strongly bind to the xCT promoter (111). BAP1 has been frequently shown to inactivate the expression of genes with mutations or deletions in tumor cells (77), but its behavior in glioma is abnormal. For example, although BAP1 is generally considered to be a chromatin-associated protein and thus to reside within the nucleus (112), recent studies have found it in both the nucleus and cytoplasm of glioma cells, suggesting BAP1 protein is differentially distributed in glioma cells (113, 114). Notably, high cytoplasmic abundance of BAP1 was significantly associated with low overall survival, and nuclear abundance of BAP1 cells was not correlated with overall survival (114). Since BAP1 shows aberrant cytosolic abundance in glioma and because the BAP1-related pathway inhibiting ferroptosis is located in the nucleus, the abnormal distribution of BAP1 in glioma cells, compared to that in other cancer cells, and the BAP1 regulatory pathway in the nucleus can be new research targets.

### OTUB1

The ubiquitin hydrolase OTUB1 was previously thought to regulate the p53 pathway by regulating the activities of Mdm2 and Mdmx (72, 73), but OTUB1 has been found to interact directly with xCT to regulate xCT independent of p53 (74). The expression of OTUB1 in glioma compared to adjacent tissues and its expression level was correlated with the low survival of glioma patients (115). Coimmunoprecipitation assays showed that the endogenous OTUB1 protein was coprecipitated with an anti-xCT-specific antibody, and endogenous xCT was coprecipitated with an anti-OTUB1-specific antibody. *In vitro* GST pull-down assays confirmed that OTUB1 is a binding partner of xCT (74). Inactivation of OTUB1 directly led to a substantial reduction in the xCT level, and this effect was confirmed to sensitize cells to erastin and the ferroptosis inhibitor ferrostatin-1 (8, 74). However, the sensitization effect caused by OTUB1 knockdown, which affected both cysteine and glutathione levels in glioma, was rescued by the overexpression of xCT (115). Notably, the ectopic overexpression of xCT is evident occurs in many cancers (68, 116–118). Hence, xCT levels may be stabilized by the absence of OTUB1, promoting ferroptosis and ultimately inhibiting tumor growth (74).

### ATF4

Activating transcription factor 4 (ATF4) is another key transcriptional regulator and mediator of metabolism and oxidative homeostasis (76, 119) that can be activated by several stress signals, such as those triggered by anoxia, hypoxia, endoplasmic reticulum (ER) stress, oxidative stress and amino acid deprivation (120). ATF4 expression is significantly higher in malignant gliomas than in untransformed human brain tissue; moreover, ATF4 can promote the proliferation and migration of glioma cells, and patients with high ATF4 expression exhibit a relatively short overall survival time (76). ATF4 expression resulted in a significant increase in xCT mRNA levels in human glioma specimens compared to that in normal brain tissue (a 5-fold increase in gliomas with a WHO° II classification and 19-fold in gliomas with a WHO° IV classification), and xCT protein levels were increased with AFT4 levels. xCT antiporter activity is determined on the basis of extracellular glutamate levels, and ATF4 knockout significantly reduced glutamate release and cystine uptake, which in turn significantly reduced xCT transporter activity (75, 76). These data suggest that ATF4 deficiency increases ROS levels in cells, but the accumulation of ROS has been shown to prevented by chelation of the iron internalized by cells, and the effects produced by ATF4 overexpression can be inhibited by sorafenib and erastin (76, 77). In addition, the growth-promoting effect of ATF4 on cells is mediated by xCT.

Pathological vessels constitute a the specific microenvironmental niche in primary brain tumors (121, 122). The expression level of ATF4 affected the growth of tumor vessels; specifically, ATF4 overexpression increased the number and length of tumor vessels, and ATF4 knockdown led to the opposite effect (76). The effects of ATF4 activity on tumor vessels were regulated by ferroptosis; moreover, erastin and RSL3 inhibited angiogenesis in glioma, and this inhibitory effect was attenuated with increased expression of ATF4 expression, although the outcome was not notable (76). ATF4 is thought to interact with components associated with ER stress (123) and to prevent cellular resistance to partial ferroptosis inducers, such as TMZ and dihydroartemisinin (124). Therefore, ATF4 is involved in multiple pathways and thus presents possibilities for ferroptosis regulation, which may lead to new research prospects.

### NCOA4

Nuclear receptor coactivator 4 (NCOA4) is a selective cargo receptor for autophagic turnover that binds to ferritin to mediate its delivery to autophagosomes and subsequently to the lysosome for ferritin degradation and concomitant iron release (78, 125, 126). When the cellular iron content is high, iron-bound NCOA4 interacts with the ubiquitin E3 ligase HERC2 to target NCOA4 for proteasomal degradation, which subsequently reduces ferritinophagy. However, when the cellular iron content is low, this interaction is inhibited, stabilizing NCOA4, which in turn increases ferritinophagic flux and iron release in lysosomes (78). This mechanism enables NCOA4 to regulate cellular iron homeostasis, determine the ferritin energy flux, and affect the sensitivity of ferroptosis-inducing agents (127–129).

Previous studies reported that NCOA4 activity led to inhibited FTH1 activity levels and that decreased FTH1 levels caused cells to respond to several ferroptosis-inducing agents, such as erastin (79, 130). Cystine deprivation led to ferroptosis, which decreased FTH1 protein levels in control glioblastoma cells (carrying NCOA4 T98G). In NCOA4-deficient GBM cells (NCOA4-knockout [KO] cells), cystine deprivation exerted little effect on the FTH1 level, and therefore, cystine removal did not cause cell death (79). Furthermore, cystine deprivation caused increases in the amount of microtubule-associated protein light chain 3 (LC3)-II (which is related to autophagosome formation) in NCOA4 T98G-mutant cells (79, 131, 132). This finding suggests that cystine deprivation induces NCOA4-mediated ferritin iron release, which in turn leads to the ferroptosis of GBM cells (79).

### YAP/TAZ

Yes-associated protein 1 (YAP) and transcriptional coactivator with PDZ-binding motif (TAZ) are two dominant effectors of the Hippo pathway. The Hippo pathway is a potent tumor suppression pathway, and its core kinases include mammalian STE20-like protein kinase 1/2 (MST1/2) and large tumor suppressor ½ (LATS1/2), which inhibit proliferation by inhibiting YAP and TAZ (133, 134). After receiving an activation signal, MST1/2 associates with Salvador 1 (SAV1) to activate the Hippo pathway and to phosphorylate LATS1/2 and its coenzyme factor MOB1. The latter then phosphorylates the transcription cofactor YAP/TAZ, and phosphorylated YAP/TAZ is isolated in the cytoplasm and not translocated to the nucleus (80). Moreover, cell density and cellular communications can influence the regulation of ferroptosis induced by YAP/TAZ (81). For example, Yang et al. showed that TAZ, but not YAP, was abundantly expressed in several cancer cell lines and underwent density-dependent nuclear translocation (135, 136). TAZ depletion led to cell resistance to various ferroptosis inducers, while overexpression of the constitutively active form of TAZ, TAZS89A, sensitized cells to ferroptosis (137).

Additionally, YAP/TAZ regulates autophagy, and overexpression of MST1/2 or contact inhibition caused by high cell density inactivates YAP/TAZ activity, suppressing the transport of autophagosome components mediated by actin-myosin complexes and reducing LC3 levels (134). In contrast, knocking down LATS1/2 activities promotes YAP/TAZ activity and autophagy, which in turn induces ferroptosis (81). Compared to that of TAZ knockdown, the inhibitory effect of YAP knockdown on ferroptosis inducers (erastin, etc.) was more significant, and the knockdown of both YAP and TAZ induced the most significant inhibitory effect (138). The expression of both YAP and TAZ was elevated in multiple tumor types, including glioma cells, and was associated with the grade of malignancy, which was highest in GBM patients (139). YAP is also regarded as an independent prognostic factor for low-grade gliomas, and studies have shown that YAP/TAZ can control GBM cell plasticity (140), which may indicate a high value for YAP and TAZ in glioma and ferroptosis research.

## Therapeutic drugs for glioma based on targeting ferroptosis

Compared with widely used ferroptosis drugs, particularly the few drugs used to treat glioma, many drugs are used to treat other malignancies, but these drugs induce drug resistance and fail to cross the blood–brain barrier, making them ineffective glioma treatments (150). TMZ is a widely used chemotherapeutic drug, but the resistance it causes is a very serious problem. Recently, research has been focused on weakening the resistance of malignant tumor cells to TMZ, and to this end, combinations of drugs and molecular hybridization are being tested (151). In addition, photodynamic therapies for ferroptosis may be used to overcome the blood–brain barrier in glioma treatment (152). Some newly tested drugs, such as dihydroartemisinin (DHA) and sulfasalazine (SAS), have shown obvious ferroptosis-inducing effects on glioma cells, and most of these drugs have been previously used to treat other malignancies. In this section, we provide an overview of the dominant therapeutic drugs used for glioma treatment that target ferroptosis. A list of these drugs is also provided in **Table 2**.

**Table 2:** Therapeutic Drugs towards Glioma Treatment by targeting Ferroptosis.

Drugs	Targets	Mechanism	Reference
DHA	GSH↓	consumes the reduced form GSH, oxidized GSSG accumulates, increases lipid ROS and MDA, inactivates GPX4 indirectly	Chen et al. (124)
TMZ	xCT↑	significantly reduced G1 phase and prolonged G2 phase	Sehm et al. (141)
	DMT1↑	broke iron homeostasis	Xue et al. (142)
		synergistically mediate the inhibition of cell activity with GPX4, Nrf2, and HO-1	Song et al. (143)
SAS	ROS↓	scavenge ROS	Aruoma et al. (144)
	ATF4↑	increase ATF4 expression, induce ER stress, decreased cell viability	Sehm et al. (145)
	xCT↓	inhibited the xCT antiporter activity hallmarked	Sehm et al. (145)
Pseudolaric acid B(PAB)	NOX4↑	activated Nox4 contributed to intracellular H2O2 and lipid peroxide and glioma cell death	Wang et al. (146)
	p53	induce GSH depletion, result in xCT inhibition	Wang et al. (146)
Ibuprofen	Nrf2↓	inhibit system xCT, inactivate GPX4 indirectly	Gao et al. (147)
Amentoflavone (AF)	FTH↓	block intracellular iron trafficking and storage to break iron homeostasis *via* modulating FTH	Chen et al. (148)
ALZ003(a curcumin analog)	AR(Androgen receptor)	induces FBXL2-mediated AR ubiquitination, leading to AR degradation then degrade GPX4	Chen et al. (149)

### Dihydroartemisinin (DHA)

Artemisinin (ART) is the active component extracted from *Artemisia annua*, and DHA, its main active derivative, has been shown to exert desired cytotoxic effects on various human malignancies (153–156).

Studies showed that the DHA-activiated pathway consumed the reduced form of glutathione (GSH) and that the oxidized form (GSSG) accumulated in glioma cells, leading to increasing levels of lipid ROS and malondialdehyde (MDA, the end product of lipid peroxidation) in glioma cells (124). In addition, transmission electron microscopy showed that the size of mitochondria was decreased, the number of mitochondrial ridges was decreased, and the bilayer membrane density was increased in DHA-treated cells, which was consistent with the ultramorphological features of cells undergoing ferroptosis (63, 157, 158). These observations also prove that DHA induced ferroptosis in glioma cells (159). To determine the targets of regulated by DHA in ferroptosis, the expression of GPX4, xCT and ACSL-4 was determined. GPX4 expression was downregulated and decreased with increasing DHA concentrations in DHA-treated groups compared to controls, while the levels of xCT and ACSL-4 were unchanged (159).

The effect of DHA on the induction of ferroptosis depended on multiple factors. Inhibition of the PERK/ATF4 signaling pathway enhanced the ferroptosis rate in DHA-induced glioma cells, and ATF4-induced HSPA5 expression was induced by increasing the GPX4 level in glioma cells undergoing DHA-induced ferroptosis (124). Thus, HSPA5 inhibitors synergistically enhanced the antitumor effects of DHA. Both the iron chelator deferoxamine (DFO) and lipid peroxidation were shown to inhibit ferrostatin-1 (Fer-1) activity, and liproxstatin-1 (Lip-1) inhibited the DHA-induced production of ROS, lipid ROS and MDA (159). Thus, both Fer-1 and Lip-1 reversed DHA-induced ferroptosis. Because DHA affects many high-impact targets and since these effects are regulated by multiple factors, studies into its selective killing effect on glioma cells are promising research directions.

### Temozolomide (TMZ)

TMZ is widely used as the first-line treatment of malignant gliomas, but its antitumor effects have not been clearly identified. Ferroptosis is considered one of the pathways targeted by TMZ, and TMZ affects ferroptosis in glioma cells in several ways. The efficacy of TMZ in human glioma depends on xCT expression, and xCT expression in cells is increased after TMZ treatment (141). TMZ induced toxicity in both xCT-silenced and xCT-overexpressing glioma cells, and the toxicity increased with increasing TMZ concentration. Significantly fewer TMZ-treated cells were found to be in the G1 or prolonged G2 phase, and xCT-silenced cells were more sensitive to TMZ than xCT-overexpressing cells (141). Astrocytes and neurons were less susceptible than glioma cells to TMZ, suggesting special implications for TMZ treatment of glioma. Moreover, the effect of TMZ was enhanced when it was combined with erastin or sorafenib (141).

TMZ induces ferroptosis through the divalent metal transporter DMT1, which regulates iron levels and maintains iron homeostasis (8, 142). Both DMT1 mRNA and protein expression levels were significantly increased in glioma cells treated with TMZ (143). When DMT1 activity was inhibited, GPX4, Nrf2, and HO-1 activity was also inhibited, and the ability of TMZ to reduce cell viability was diminished (143). These results suggest that TMZ induces the ferroptosis of glioma cells and that this effect was associated with xCT and DMT1 expression.

### Sulfasalazine (SAS)

SAS has been shown to scavenge ROS (144), induce cancer apoptosis (160), and attenuate glioma-induced epilepsy (161, 162). Recent studies showed that SAS significantly increased ATF4 expression in glioma cells and induced ER stress, decreasing cell viability (145). Cell death was prevented by treatment with iron chelators and ferroptosis inhibitors, and high concentrations of SAS specifically inhibited the expression of an xCT antiporter activity marker (145), confirming that high concentrations of SAS inhibited xCT activity and induced ferroptosis in glioma cells. In experiments with a rat model, SAS significantly reduced glioma cell proliferation, exerted no significant toxic effects on normal neurons (163) and mild toxicity on astrocytes, and did not affect brain cell viability (145). However, due to low brain penetration, SAS showed poor efficacy in newly diagnosed and recurrent malignant glioma (150, 164). This problem is expected to be improved by convection-enhanced delivery (CED) (163).

In addition, SAS is likely to be used in several drug combinations. For example, molecular hybridization product of SAS and DHA, called AC254, showed significantly higher effects on glioma cells than either drug administered separately or in other drug combinations (165). AC254 led to changes in glioma cell shape and activity and terminated cell division, which were significantly better outcomes than those induced by the parent drugs and their mixture with other drugs (165). SAS enhanced the ability of TMZ to reduce human GBM cell activity (151), which may solve the problem of TMZ resistance.

## Conclusions and perspectives

As a recently discovered form of cell death, ferroptosis shows many potential applications to glioma treatment. Recent studies have revealed three major pathways of ferroptosis, namely, iron metabolism, lipid peroxidation, and antioxidant system pathways (26). Ferroptosis is primarily regulated by the inhibition of xCT, accumulation of ROS, inhibition of GPX and GSH, which are mediators of many secondary regulatory pathways. In addition to these findings, increasing evidence links ferroptosis with autophagy, which has led to multiple research directions (166). The regulatory pathways of ferroptosis and the relationship of these pathways between ferroptosis and other forms of cell death remain to be further investigated.

Glioma cells show sensitivity to multiple types of specific ferroptosis inducers. Several critical factors inducing ferroptosis show different degrees of abnormal manifestation in glioma cells; for example, GPX4, Nrf2 and ATF4 show high expression compared with normal cells, and p53 shows complex regulatory effects. These findings provide therapeutic targets for glioma. However, few studies have focused on the specific activities of ferroptosis-related factors in glioma, and to identify more factors and their complex roles, more experiments need to be conducted.

Ferroptosis provides potential targets for further glioma treatment. Due to the complex regulatory mechanism of ferroptosis, many drugs show completely different effects *in vivo* than *in vitro* or show varying degrees of antagonistic effects in different pathways. In summary, the specific mechanism of ferroptosis remains unclear, and the indicators of ferroptosis are not obvious. Therefore, research on ferroptosis-related drugs needs to be conducted based on information obtained through additional detailed studies.

## Data availability statement

The original contributions presented in the study are included in the article/supplementary material. Further inquiries can be directed to the corresponding authors.

## Author contributions

YZ, CF, and HX designed the review and wrote the manuscript. YL, XW, and LY conceived the artwork and performed the bibliographical research. AZ, AS, and DZ supervised the writing. All the authors revised and approved the final version of the manuscript.

## Funding

This work was supported by grants from the Project Supported by Zhejiang Provincial Natural Science Foundation of China (LY22H090020).

## Conflict of Interest

The authors declare that the research was conducted in the absence of any commercial or financial relationships that could be construed as a potential conflict of interest.

## Publisher’s note

All claims expressed in this article are solely those of the authors and do not necessarily represent those of their affiliated organizations, or those of the publisher, the editors and the reviewers. Any product that may be evaluated in this article, or claim that may be made by its manufacturer, is not guaranteed or endorsed by the publisher.
